# Hopf bifurcation analysis of a delayed SEIR epidemic model with infectious force in latent and infected period

**DOI:** 10.1186/s13662-018-1805-6

**Published:** 2018-10-01

**Authors:** Aekabut Sirijampa, Settapat Chinviriyasit, Wirawan Chinviriyasit

**Affiliations:** Department of Mathematics, Faculty of Science, King Mongkut’s University of Technology Thonburi, Bangkok, Thailand

**Keywords:** *SEIR* epidemic model, Time delay, Standard incidence, Hopf bifurcation

## Abstract

In this paper, we analyze a delayed *SEIR* epidemic model in which the latent and infected states are infective. The model has a globally asymptotically stable disease-free equilibrium whenever a certain epidemiological threshold, known as the basic reproduction number $R_{0}$, is less than or equal to unity. We investigate the effect of the time delay on the stability of endemic equilibrium when $R_{0}>1$. We give criteria that ensure that endemic equilibrium is asymptotically stable for all time delays and a Hopf bifurcation occurs as time delay exceeds the critical value. We give formulae for the direction of Hopf bifurcations and the stability of bifurcated periodic solutions by applying the normal form theory and the center manifold reduction for functional differential equations. Numerical simulations are presented to illustrate the analytical results.

## Introduction

Since the pioneering work of Kermack and McKendrick [1] on compartment modeling, mathematical modeling has become an important tool in analyzing the spread and control of infectious diseases. Recently, great attention has been paid to developing realistic mathematical models for the transmission dynamics of infectious diseases, such as the severe acute respiratory syndromes (SARS) outbreak in 2003 [2, 3], the avian influenza A (H7N9) outbreak in China in 2013 [4, 5], and potential mechanisms behind the spread of AH1N1 influenza virus in different regions around the world [6].

Delays play an important role in the dynamics of populations. In many real-world processes, especially, in a lot of biological phenomena, the present dynamics of the state variables depends not only on the present state of the processes but also on the history of the phenomenon, that is, on the past values of state variables. The time delay may influence the dynamics of infectious diseases. In fact, many diseases have different kinds of delays when they spread, such as immunity period delay [7, 8], infection period delay [9], and incubation period delay [10–14]. It is well known that the dynamical behaviors (including stability, attractivity, persistence, periodic oscillation, bifurcation, and chaos) of population models with time delay have become a subject of intense research activities. In particular, the properties of periodic solutions arising from the Hopf bifurcation are of great interest. A number of epidemic models with time delay have been developed in the literature to gain insights into the effect of time delay on the dynamic behavior of the model (see, e.g., [15–27]). Li et al. [15] investigated the existence of a positive solution and local stability for the steady state of an age-structured *SEIR* epidemic model. Röst and Wu [16] analyzed the global stability of an *SEIR* model with distributed infinite time delay when the infectivity depends on the age of infection. Gao et al. [17] formulated an *SEIR* epidemic model with two time delays and pulse vaccination for studying the control of spread and transmission of an infectious disease. Tipsri and Chinviriyasit [27] investigated the effect of time delay on the stability of bifurcating periodic solutions and direction of Hopf bifurcation of an *SEIR* model with nonlinear incidence.

In addition, the course of an epidemic depends on the contact rate between susceptible and infected individuals and on the assumption that the net rate at which infections are acquired is proportional to the number of encounters between susceptible and infected individuals denoted by *S* and *I*, respectively. The constant of proportionality *β* is sometimes called the transmission coefficient [28]. This transmission coefficient may well depend on the population size. If the total population size *N* is not too large, then the bilinear incidence, denoted by $\beta S I$, is proper for the model because the number of adequate contacts by an individual per unit time should increase as the total population size *N* increases. On the other hand, if the population size *N* is quite large, then the standard incidence, denoted by $\beta S I/N$, is more realistic [29]. These two incidences are widely used in modeling the transmission dynamics of the human diseases [13, 28, 30]. Thus, the formulation of the incidence function is an important aspect of the mathematical study of epidemiology.

In view of the above, the aim of this paper is to formulate and analyze a delayed *SEIR* epidemic model, in which the latent and infected states are infective, for the occurrence of Hopf bifurcation. The paper is organized as follows. In Sect. 2, we present a delayed *SEIR* epidemic model with infectious force in latent and infected periods and give the basic properties of the model. The local and global asymptotic stabilities of disease-free equilibrium are established in Sect. 3. The local stability of the endemic equilibrium and sufficient and necessary conditions for the existence of the Hopf bifurcation are analyzed in Sect. 4. In Sect. 5, when the model exhibits the Hopf bifurcation, we employ the normal form theory and center manifold approach to derive formulas for determining the direction and stability of bifurcating periodic solutions. Numerical simulations are carried out in Sect. 6 to illustrate the main theoretical results, and a brief discussion is given in Sect. 7 to conclude this work.

## Model formulation and basic properties

The delayed model is formulated under the following assumptions. The four-dimensional model, at time *t*, monitors the dynamics of the susceptible individuals $S(t)$, exposed individuals $E(t)$, infectious individuals $I(t)$, and recovered individuals $R(t)$, respectively. Thus the total population at time *t* is $N(t)=S(t)+E(t)+I(t)+R(t)$. People who have been infected first go into a latent (exposed) stage, during which they may have a low level of infectivity, then susceptible individuals may infect from both exposed and infectious individuals at the rates $\beta\beta_{E}$ and $\beta\beta_{I}$, respectively. The parameters *β*, $\beta_{E}$, and $\beta_{I}$ denote the contact rate, the ability to cause infection by exposed individuals $(0\leq\beta_{E}\leq 1)$ and infection by infected individuals $(0\leq\beta_{I}\leq 1)$. We assume that on adequate contact with an infective, a susceptible individual is exposed at a time $t-\tau$ and becomes infective (assumed to be infectious) a time *τ* later. Taking these assumptions, the delay differential equations are given by

2.1 $$\begin{aligned} \begin{aligned}& \frac{dS(t)}{dt}=\Pi-\frac{\beta\beta_{E} E(t-\tau )+\beta\beta_{I} I(t-\tau)}{N(t-\tau)}S(t-\tau)-\mu S(t), \\ &\frac{dE(t)}{dt}=\frac{\beta\beta_{E} E(t-\tau)+\beta \beta_{I} I(t-\tau)}{N(t-\tau)}S(t-\tau)-(\mu+\sigma+\kappa )E(t), \\ &\frac{dI(t)}{dt}=\sigma E(t)-(\mu+\alpha+\gamma)I(t), \\ &\frac{dR(t)}{dt}=\kappa E(t)+\gamma I(t)-\mu R(t), \end{aligned} \end{aligned}$$

where Π is the recruitment rate (by birth or immigration) into the population (assumed susceptible), *μ* is the natural death rate, *σ* is the rate at which exposed individuals become infectious, *κ* is the recovery rate of exposed individuals, *γ* is the recovery rate of infected individuals, and *α* is the rate of disease-induced death. For model (2.1), we also assume that infected individuals who are effectively treated move into the recovery class by achieving temporary immunity against the disease. It should be noted that since the model monitors human populations, all the model parameters and variables are assumed to be nonnegative.

The initial condition of (2.1) is given by

2.2 $$\begin{aligned} \begin{aligned}& S(\theta)=\phi_{1}(\theta),\qquad E(\theta)= \phi_{2}(\theta),\qquad I(\theta )=\phi_{3}(\theta),\\ & R(\theta)= \phi_{4}(\theta),\quad \theta\in[-\tau,0], \end{aligned} \end{aligned}$$

with $\phi=[\phi_{1}, \phi_{2}, \phi_{3}, \phi_{4}]\in\mathbb{C}$ such that $\phi_{i}(\theta)=\phi_{i}(0)\geq0$, $i= 1,2,3,4$, for $\theta\in[-\tau,0]$, where $\mathbb{C}$ denotes the Banach space of continuous functions mapping the interval $[-\tau,0]$ into

$$\begin{aligned} \mathbb{R}^{4}_{+}=\bigl\{ (S,E,I,R): S\geq0, E\geq0, I\geq0, R\geq0\bigr\} . \end{aligned}$$

It is well known by the fundamental theory of functional differential equations [31] that system (2.1) has a unique solution $(S(t),E(t),I(t),R(t))$ satisfying the initial conditions (2.2). It can be shown (see [32] for more detail) that all solutions of system (2.1) with initial conditions (2.2) are defined on $[0,\infty)$ and remain positive for all $t\geq0$.

Further, it is easy to show that the system has two positive equilibriums, namely:

(i) *Disease-free equilibrium* (*DFE*) $\mathcal{E}_{0}= ( \frac{\Pi}{\mu},0,0,0 )$;
(ii) *Endemic equilibrium*(*EE*) $\mathcal{E}^{*}= (S^{*},E^{*},I^{*},R^{*} )$, where
  2.3 $$ \begin{aligned} &S^{*}=\frac{\Pi(k_{1} k_{2}-\sigma\alpha)}{\mu(k_{1} k_{2} R_{0}-\sigma\alpha)},\qquad E^{*}=\frac{k_{2}\Pi(R_{0}-1)}{k_{1} k_{2} R_{0}-\sigma\alpha}, \\ & I^{*}=\frac{\sigma\Pi(R_{0}-1)}{k_{1} k_{2} R_{0}-\sigma\alpha}, \qquad R^{*}=\frac{\Pi(\kappa k_{2}+\sigma\gamma )(R_{0}-1)}{\mu(k_{1} k_{2} R_{0}-\sigma\alpha)} \end{aligned} $$

with $k_{1}= \mu+\kappa+\sigma$, $k_{2}= \mu+\gamma+\alpha$, $\beta_{1} = \beta\beta_{E}$, $\beta_{2} = \beta\beta_{I}$, and $R_{0}= \frac{\beta_{1} k_{2} +\beta_{2} \sigma}{k_{1} k_{2}}$. It follows from (2.3) that a sufficient and necessary condition for the existence of endemic equilibrium is $R_{0}>1$, that is, the infection is maintained in the population. The threshold quantity $R_{0}$ is called the basic reproduction number of model (2.1) [28].

To discuss the local stability of equilibria, we first give the following definition of stability types.

### Definition 2.1

([27])

The positive equilibrium is absolutely stable if it is asymptotically stable for every delay $\tau\geq0$ and is conditionally stable if it is asymptotically stable for *τ* in some finite interval.

## Local and global stability of disease-free equilibrium

For the local stability of a disease-free equilibrium, we claim the following theorem.

### Theorem 3.1

*The disease*-*free equilibrium* $\mathcal{E}_{0}$ *of model* (2.1) *is*

(i) *absolutely stable if* $R_{0}<1$,
(ii) *linearly neutrally stable if* $R_{0}=1$, *and*
(iii) *unstable if* $R_{0} > 1$.

### Proof

By linearization the Jacobian of system (2.1) evaluated at $\mathcal{E}_{0}$ is given by

$$J(E_{0})=\left[\begin{array}{cccc} -\mu & -\beta_{1}e^{-\lambda \tau } & -\beta_{2}e^{-\lambda \tau } & 0\\ 0 & -k_{1}+\beta_{1}e^{-\lambda \tau } & \beta_{2}e^{-\lambda \tau } & 0\\ 0 & \sigma & -k_{2} & 0\\ 0 & \kappa & \gamma & -\mu \end{array}\right],$$

with eigenvalues $\lambda_{1} = \lambda_{2} = -\mu$ and the roots of the transcendental polynomial

3.1 $$ f(\lambda)= \lambda^{2}+ (k_{1}+k_{2} )\lambda +k_{1}k_{2}- (\beta_{1} \lambda+k_{1} k_{2} R_{0} )e^{-\lambda\tau}=0. $$

For $\tau=0$, (3.1) reduces to

3.2 $$ f(\lambda)= \lambda^{2}+ \biggl(k_{2}+\frac{\sigma\beta _{2}}{k_{2}}+k_{1}(1-R_{0}) \biggr)\lambda +k_{1}k_{2}(1-R_{0})=0. $$

It is easy to see that if $R_{0} <1$, then the roots of (3.2) have negative real parts. Thus the disease-free equilibrium is locally asymptotically stable when $\tau=0$.

For $\tau>0$, let $\lambda=i\omega(\omega>0)$ be the root of (3.1). After substituting and separating into real and imaginary parts, we have

3.3 $$ \begin{aligned}& \beta_{1}\omega\sin\omega \tau+k_{1} k_{2} R_{0} \cos\omega\tau = - \omega^{2} +k_{1}k_{2}, \\ &{-}k_{1} k_{2} R_{0}\omega\sin\omega\tau+ \beta_{1}\omega\cos\omega\tau = (k_{1} + k_{2}) \omega, \end{aligned} $$

which implies

3.4 $$ \omega^{4}+a_{1}\omega^{2}+a_{0}=0, $$

where

$$\begin{aligned} &a_{0} = k_{1}^{2}k_{2}^{2} \bigl(1-R_{0}^{2}\bigr), \\ &a_{1} = k_{2}^{2}+(k_{1}+ \beta_{1}) \biggl(\frac{\beta_{2}\sigma }{k_{2}}+k_{1}(1-R_{0}) \biggr). \end{aligned}$$

Since $a_{0}$ and $a_{1}$ are positive whenever $R_{0} < 1$, it follows that $\omega^{2}$ is negative. The contradiction shows that (3.1) has no purely imaginary root for $\tau>0$. Hence, by Definition 2.1 and Lemma 3.5(i) [27], the disease-free equilibrium $\mathcal{E}_{0}$ is absolutely stable for $\tau\geq0$.

If $R_{0}=1$, then the transcendental polynomial (3.1) becomes

3.5 $$ \lambda^{2}+ (k_{1}+k_{2} )\lambda +k_{1}k_{2}- (\beta_{1} \lambda+k_{1}k_{2} )e^{-\lambda\tau}=0. $$

It is clear that $\lambda= 0$ is a simple root of (3.5). We will further show that any root of (3.5) must have a negative real part except $\lambda= 0$. In fact, (3.5) has imaginary roots as $\lambda=u\pm{\rm i} \omega$ for some $u \geq0$, $\omega\geq0$, and $\tau\geq0$. It follows from (3.5) that

$$\begin{aligned} &{u}^{2}-{\omega}^{2}+(k_{{2}}+k_{{1}})u+k_{{1}}k_{{2}} = \bigl( ( \beta_{{1}}u+k_{{1}}k_{{2}} ) \cos ( \omega \tau ) - \beta_{{1}}\omega \sin ( \omega\tau ) \bigr) {e}^{- u\tau} , \\ &\omega ( k_{{2}}+k_{{1}}+2 u ) = \bigl( ( \beta_{{ 1}}u+k_{{1}}k_{{2}} ) \sin ( \omega\tau ) + \beta_{{ 1}}\omega \cos ( \omega\tau ) \bigr) {e}^{- u\tau}, \end{aligned}$$

which, together with $u \geq0$, implies that

$$\begin{aligned} &\bigl( {u}^{2}+2 k_{{2}}u+{\omega}^{2}+{k_{{2}}}^{2} \bigr) \bigl( {u}^{2}+2 k_{{1}}u+{\omega}^{2}+{k_{{1}}}^{2} \bigr) \\ &\quad= \bigl[ ( \beta_{{1}}u+k_{{1}}k_{{2}} ) ^{2}+{\beta_{{1}}}^{2}{ \omega}^{2} \bigr]{e}^{-2 u\tau} \\ &\quad\leq ( \beta_{{1}}u+k_{{1}}k_{{2}} ) ^{2}+{\beta_{{1}}}^{2}{ \omega}^{2}. \end{aligned}$$

It is easy to check that this inequality is not true. This shows that any root of (3.5) has a negative real part except $\lambda= 0$, which implies that $\mathcal{E}_{0}$ is linearly neutrally stable when $R_{0} = 1$.

In the case where $R_{0} > 1$, from (3.1) it follows that $f(0)<0$ and $\lim_{\lambda\to\infty} f(\lambda)=+\infty$. From the continuity of the function $f(\lambda)$ on $(-\infty,\infty)$ it follows that the transcendental equation (3.1) has at least one positive real root. Hence $\mathcal{E}_{0}$ is unstable. Therefore the theorem is proved. □

Define the region

$$\begin{aligned} \Omega=\bigl\{ (S,E,I,R) \in\mathbb{R}^{4}_{+}: S+E+I+R \leq \Pi/\mu\bigr\} . \end{aligned}$$

Adding all equations in (2.1) gives $dN/dt = \Pi/\mu- \alpha I$. Consequently, in the absence of infection, $N \to \Pi/\mu$ as $t \to\infty$, and $\Pi/\mu$ is an upper bound of $N(t)$, provided that $N(0)\leq\Pi/\mu$. Also, if $N(0) > \Pi/\mu$, then *N* will decrease to this level. Thus Ω is positively invariant with respect to system (2.1). The global stability of the disease-free equilibrium is therefore established in the following theorem.

### Theorem 3.2

*The disease*-*free equilibrium* $\mathcal{E}_{0}$ *of system* (2.1) *is globally asymptotically stable in* Ω *if* $R_{0}\leq1$.

### Proof

Let $x_{t}$ represent the translation of the solution of system (2.1) with initial conditions (2.2), that is, $x_{t} = (S(t+\theta),E(t+\theta),I(t+\theta),R(t+\theta))$, and $N(t+\theta)=S(t+\theta)+E(t+\theta)+I(t+\theta)+R(t+\theta)$ for $\theta\in[0,\infty)$. We introduce the Lyapunov function

3.6 $$ V(x_{t})=\frac{\beta_{1}k_{2}+\beta_{2}\sigma}{k_{1}k_{2}}E+\frac {\beta_{2}}{k_{2}}I + \frac{\beta_{1}k_{2}+\beta_{2}\sigma}{k_{1}k_{2}} \int_{t-\tau }^{t} \biggl(\frac{\beta_{1}E(\theta)+\beta_{2}I(\theta)}{N(\theta )}S(\theta) \biggr) \,d\theta. $$

Note that $V\geq0$ along the solutions of system (2.1). In addition, $V=0$ if and only if both *E* and *I* are zero. The derivative of *V* along the solutions of system (2.1) is given by

$$\begin{aligned} \frac{dV}{dt}={}&\frac{\beta_{1}k_{2}+\beta_{2}\sigma }{k_{1}k_{2}} \biggl( \frac{\beta_{1}E(t-\tau)+\beta_{2}I(t-\tau )}{N(t-\tau)}S(t-\tau)-k_{1}E(t) \biggr) +\frac{\beta_{2}}{k_{2}} \bigl(\sigma E(t)-k_{2}I(t) \bigr) \\ &{}+\frac{\beta_{1}k_{2}+\beta_{2}\sigma}{k_{1}k_{2}} \biggl(\frac {\beta_{1}E(t)+\beta_{2}I(t)}{N(t)}S(t) \biggr) \\ &{}-\frac{\beta_{1}k_{2}+\beta_{2}\sigma}{k_{1}k_{2}} \biggl(\frac {\beta_{1}E(t-\tau)+\beta_{2}I(t-\tau)}{N(t-\tau)}S(t-\tau) \biggr) \\ = {}&\biggl(\frac{\beta_{1}k_{2}+\beta_{2}\sigma}{k_{1}k_{2}} \biggr) \frac{(\beta_{1}E(t)+\beta_{2}I(t))S(t)}{N(t)}-(\beta_{1}E+ \beta_{2} I) \\ ={}&(\beta_{1}E+\beta_{2} I) \biggl(R_{0}\frac{S(t)}{N(t)}-1 \biggr) \\ \leq{}&{-}(\beta_{1}E+ \beta_{2}I) (1-R_{0}). \end{aligned}$$

Thus $dV/dt < 0$ if $R_{0} < 1$, whereas $dV/dt=0$ if and only if $R_{0} = 1$ or $E=I=0$. Consequently, the maximum invariance set in $\{(S,E,I,R)\in\Omega:dV/dt=0\}$ when $R_{0} \leq1$ is the singleton {${\mathcal{E}_{0}}$}. Therefore, the LaSalle’s invariance principle [31] implies that $\mathcal{E}_{0}$ is globally asymptotically stable in Ω. This proves the theorem. □

## Hopf bifurcation analysis

In this section, we determine sufficient and necessary conditions for Hopf bifurcation to occur using the time delay *τ* as the bifurcation parameter. In this section, we assume that $R_{0}>1$, that is, the endemic equilibrium $\mathcal{E}^{*}$ exists. To study the stability of $\mathcal{E}^{*}$, we consider the linearization of system (2.1) at the point $\mathcal{E}^{*}$. The corresponding transcendental characteristic equation is given by

4.1 $$ (\lambda+\mu) \bigl(\lambda^{3}+a_{2} \lambda^{2}+a_{1}\lambda+a_{0}+\bigl(b_{2} \lambda ^{2}+b_{1}\lambda+b_{0}\bigr)e^{-\lambda\tau} \bigr)=0, $$

where

$$\begin{aligned} &a_{0}=\mu k_{1}k_{2},\qquad a_{1}=k_{1}k_{2}+\mu(k_{1}+k_{2}),\qquad a_{2}=\mu+k_{1}+k_{2},\qquad Q^{*}=\frac{\beta_{1} E^{*}+\beta_{2} I^{*}}{N^{*}} \\ &b_{0}=-\mu k_{{1}}k_{{2}}+k_{{1}}k_{{2}}Q^{*}- {\frac{\sigma \alpha Q^{*}}{R_{{0} }}},\qquad b_{1}=-k_{{1}}k_{{2}}-{ \frac{\beta_{1} \mu}{R_{{0}}}}+ ( k_{{1}}+ k_{{2}} ) Q^{*},\\ & b_{2}=Q^{*}-{\frac{\beta_{1}}{R_{{0}}}}. \end{aligned}$$

For the case $\tau=0$, (4.1) is rewritten as

4.2 $$ (\lambda+\mu) \bigl(\lambda^{3}+c_{1} \lambda^{2}+c_{2}\lambda+c_{0}\bigr)=0, $$

where

$$\begin{aligned} &c_{2} = \mu+k_{2}+\frac{\beta_{2}\sigma}{R_{0} k_{2}}+Q^{*}>0, \\ &c_{1} = \mu k_{2}+Q^{*}(k_{1}+k_{2})+\frac{\mu\beta_{2}\sigma}{R_{0} k_{2}}>0, \\ &c_{0} = \frac{Q^{*}}{R_{0}} \bigl(\sigma\alpha(R_{0}-1)+R_{0} \bigl[k_{2}(\mu+\kappa)+\sigma(\mu+\gamma) \bigr] \bigr)>0. \end{aligned}$$

Then we see that

$$\begin{aligned} c_{1}c_{2}-c_{0} = {}& \biggl(\mu+Q^{*}+\frac{\beta_{2}\sigma}{R_{0}k_{2}} \biggr) \biggl(\mu k_{2}+Q^{*}(k_{1}+k_{2})+ \frac{\mu\beta_{2}\sigma}{R_{0} k_{2}} \biggr) +k_{2} \biggl(\mu k_{2}+Q^{*} k_{2}+\frac{\mu\beta_{2}\sigma}{R_{0} k_{2}} \biggr) \\ &{}+\frac{\sigma\alpha Q^{*}}{R_{0}} >0. \end{aligned}$$

Thus, by the Routh–Herwitz criterion, all roots of (4.2) are negative, which means that the endemic equilibrium $\mathcal{E}^{*}$ is locally asymptotically stable in the case $\tau=0$.

Next, we will investigate the distribution of positive roots of the equation

4.3 $$ \lambda^{3}+a_{2}\lambda^{2}+a_{1} \lambda+a_{0}+\bigl(b_{2}\lambda^{2}+b_{1} \lambda +b_{0}\bigr)e^{-\lambda\tau}=0. $$

For $\tau>0$, *iω* $(\omega>0)$ is a root of (4.3) if and only if *ω* satisfies

$$ -\omega^{3}\text{i}-a_{2}\omega^{2}+a_{1} \omega \text{i}+ a_{0}+\bigl(-b_{2}\omega ^{2}+b_{1} \omega \text{i}+b_{0}\bigr) (\cos\omega\tau-\text{i}\sin\omega\tau)=0. $$

Separating the real and imaginary parts, we have

4.4 $$\begin{aligned} &\bigl(-b_{2}\omega^{2}+b_{0}\bigr)\cos\omega \tau+b_{1}\omega\sin\omega\tau = a_{2}\omega^{2}-a_{0}, \end{aligned}$$

4.5 $$\begin{aligned} &b_{1}\omega\cos\omega\tau+\bigl(b_{2}\omega^{2}-b_{0} \bigr)\sin\omega\tau = \omega^{3}-a_{1}\omega, \end{aligned}$$

which implies

4.6 $$ z^{3}+pz^{2}+qz+r=0, $$

where

$$\begin{aligned} &z = \omega^{2}, \\ &p = a_{2}^{2}-b_{2}^{2}-2a_{1}, \\ &q = a_{1}^{2}-b_{1}^{2}+2b_{0}b_{2}-2a_{0}a_{2}, \\ &r = a_{0}^{2}-b_{0}^{2}. \end{aligned}$$

To investigate the local stability of endemic equilibrium $\mathcal{E}^{*}$, we first assume that the following conditions hold:

1. $\beta_{1} k_{2} +\beta_{2} \sigma-k_{1} k_{2} >0$; then the disease-free equilibrium does not exist,
2. $\beta_{1} k_{2} +\beta_{2} \sigma- \sigma\alpha>0$,
3. $Q^{*} \ll1$.

Thus, when $R_{0}>1$ and (H1)–(H3) hold, we have

$$\begin{aligned} p = {}& \frac{k_{{1}}\beta_{{2}}\sigma}{k_{2} R_{0}} \biggl( 1+{\frac {\beta_{{1}}}{k_{{1}}R_{{0} }}} \biggr) +{ \frac{2q\beta_{{1}}}{R_{{0}}}}+ \mu^{2} + \bigl(k_{2}+Q^{*}\bigr) \bigl(k_{2}-Q^{*}\bigr), \\ q ={}& \frac{2 Q^{*}\beta_{{1}}}{R_{0}} \biggl[\mu ( k_{{1}}+k_{{2}} ) +{ \frac{\sigma \alpha}{R_{{0}}}} \biggr]+\mu^{2} k^{2}_{2} \biggl[1+\frac{k_{1} \beta_{2} \sigma}{k_{2}^{3} R_{0}} \biggl( 1+\frac{\beta_{1}}{k_{1} R_{0}} \biggr) \biggr] \\ &{}+ \frac{2 Q^{*} k_{{1}}\beta_{{2}}\sigma}{R_{0}} \biggl( 1-{\frac {Q^{*}\alpha}{k_{{1}}\beta_{{2}}}} \biggr) \\ &{}+2 k_{1} k_{2} (\gamma+\alpha) \biggl(1-\frac{Q^{*} ( k^{2}_{1}+k_{2}^{2} ) }{2k_{1} k_{2}( \gamma+\alpha) } \biggr), \\ r ={}& {\frac{{\mu}^{2}k_{1}^{2} k_{2}^{2} ( R_{{0}}-1 ) ( \beta_{1} k_{{2}}+\beta_{2} \sigma-\sigma\alpha ) }{ ( k_{{1}}k_{{2}}-\sigma\alpha ) ^{2}{R_{{0}}} ^{2}}} \bigl[ ( 3 k_{{1}}k_{{2}}- \sigma\alpha ) R_{{0}}- \bigl(k_{{1}}k_{{2 }}R_{0}^{2}+ \sigma\alpha\bigr) \bigr]. \end{aligned}$$

It follows that *p* and *q* are positive, whereas *r* is positive or negative depending on the condition. Thus we consider two cases.

*Case* 1. Rewriting *r* as

4.7 $$\begin{aligned} r=R_{{0}} ( 2 k_{1} k_{2} -\sigma \alpha-k_{1} k_{2} R_{0} ) +(\beta_{1} k_{2} + \beta_{2} \sigma-\sigma\alpha), \end{aligned}$$

we see that $r>0$ if the following condition holds:

$$\begin{aligned} 1< R_{0} < 1+\frac{k_{1} k_{2} -\sigma\alpha}{k_{1} k_{2}}. \end{aligned}$$

This gives the condition for the contact rate *β*:

4.8 $$\begin{aligned} \frac{k_{1} k_{2}}{\beta_{E} k_{2} + \beta_{I} \sigma}< \beta< \frac{2 k_{1} k_{2} -\sigma\alpha}{\beta_{E} k_{2} + \beta_{I} \sigma}. \end{aligned}$$

Since $p,q, r > 0$, (4.3) has no positive real roots. Moreover,

$$\begin{aligned} a_{1} a_{2}-a_{0} = ( k_{{1}}+k_{{2}} ) ( \mu+k_{{2}} ) ( \mu+k_{{1}} )>0. \end{aligned}$$

Therefore by Lemma 3.4(a) and Lemma 3.5(i) in [27] we obtain the following theorem.

### Theorem 4.1

*If* $R_{0} > 1$ *and condition* (4.8) *holds*, *then the endemic equilibrium* $\mathcal{E}^{*}$ *of model* (2.1) *is absolutely stable for* $\tau\geq0$.

*Case* 2. Rewriting *r* as

$$\begin{aligned} r=-R_{{0}} \bigl[ k_{{1}}k_{{2}}R_{{0}}-(3k_{{1}}k_{{2}}- \sigma \alpha) \bigr] -\sigma\alpha, \end{aligned}$$

we see that $r<0$ if the following condition holds:

$$\begin{aligned} R_{{0}} > 2+\frac{k_{1} k_{2}-\sigma\alpha}{k_{1} k_{2}}. \end{aligned}$$

This gives the following condition for the contact rate *β*:

4.9 $$ \beta> \frac{3k_{1} k_{2}-\sigma \alpha}{\beta_{E}k_{2}+\beta_{I}\sigma}. $$

By Lemma 3.3(c) [27], (4.3) has positive real roots, that is, the characteristic equation (4.6) has a pair of purely imaginary roots of the form $\lambda=\pm i\omega_{0}$.

Substituting $\omega=\omega_{0}$ into (4.4)–(4.5) and solving for *τ*, we get the corresponding $\tau_{n}>0$, $n=0,1,2,\dots$:

4.10 $$ \tau_{n} = \frac{1}{\omega_{0}}\cos^{-1} \biggl\{ \frac{(\omega _{0}^{3}-a_{1}\omega_{0})b_{1}\omega_{0}-(a_{2}\omega_{0}^{2}-a_{0})(b_{2}\omega _{0}^{2}-b_{0})}{(b_{0}-b_{2}\omega_{0}^{2})^{2}+(b_{1}\omega_{0})^{2}} \biggr\} +\frac {2\pi n}{\omega_{0}}. $$

By Lemma 3.4(c) in [27] all roots of (4.3) have negative real parts for $\tau\in[0,\tau_{0})$. Therefore, by Lemma 3.5(ii) [27], we obtain the following theorem.

### Theorem 4.2

*If* $R_{0} > 1$ *and condition* (4.9) *holds*, *then the endemic equilibrium* $\mathcal{E}^{*}$ *of model* (2.1) *is conditionally stable for* $\tau\in [0,\tau_{0})$.

For the bifurcation analysis, the time delay *τ* is chosen as the bifurcation parameter, and we will show that there exists at least one eigenvalue with positive real part for $\tau> \tau_{0}$, that is, $\frac{d(\operatorname{Re}\lambda)}{d\tau} \vert _{\tau=\tau_{0}}>0$.

The derivative of (4.3) with respect to *τ* is given by

$$\begin{aligned} &(\lambda+\mu) \biggl\{ \bigl(3\lambda^{2}+2a_{2} \lambda+a_{1}\bigr)\frac{d\lambda }{d\tau}- \bigl(b_{2} \lambda^{2}+b_{1}\lambda+b_{0}\bigr)e^{-\lambda\tau} \biggl(\lambda+\tau \frac{d\lambda}{d\tau} \biggr) \\ &\quad{}+e^{-\lambda\tau}(2b_{2} \lambda+b_{1})\frac{d\lambda}{d\tau} \biggr\} \\ &\quad{}+ \bigl(\lambda^{3}+a_{2}\lambda^{2}+a_{1} \lambda+a_{0}+\bigl(b_{2}\lambda^{2} +b_{1}\lambda+b_{0}\bigr)e^{-\lambda\tau} \bigr) \frac{d\lambda}{d\tau}=0. \end{aligned}$$

After rearranging this equation, we get

$$\begin{aligned} \biggl(\frac{d\lambda}{d\tau} \biggr)^{-1} = \frac{2\lambda^{3}+a_{2}\lambda^{2}-a_{0}}{-\lambda^{2}(\lambda ^{3}+a_{2}\lambda^{2}+a_{1}\lambda+a_{0})}+\frac{b_{2}\lambda^{2}-b_{0}}{\lambda ^{2}(b_{2}\lambda^{2}+b_{1}\lambda+b_{0})}-\frac{\tau}{\lambda}. \end{aligned}$$

Therefore

$$\begin{aligned} &\operatorname{sign} \biggl\{ \frac{d(\operatorname{Re}\lambda)}{d\tau} \biggr\} _{\tau=\tau_{0}} \\ &\quad= \operatorname{sign} \biggl\{ \operatorname{Re} \biggl(\frac{d\lambda }{d\tau} \biggr)^{-1}_{\lambda=\text{i}\omega_{0}} \biggr\} \\ &\quad= \operatorname{sign} \biggl\{ \frac{(a_{2}\omega^{2}+a_{0})(a_{2}\omega ^{2}-a_{0})-2\omega^{3}(a_{1}\omega-\omega^{3})+(b_{0}+b_{2}\omega ^{2})(b_{0}-b_{2}\omega^{2})}{\omega^{2}[(b_{0}-b_{2}\omega^{2})^{2}+(b_{1}\omega )^{2}]} \biggr\} \\ &\quad= \operatorname{sign} \biggl\{ \frac{-(a_{0}^{2}-a_{2}^{2}\omega^{4})-2a_{1}\omega ^{4}+2\omega^{6}+b_{0}^{2}-b_{2}^{2}\omega^{4}}{\omega^{2}[(b_{0}-b_{2}\omega ^{2})^{2}+(b_{1}\omega)^{2}]} \biggr\} \\ &\quad = \operatorname{sign} \biggl\{ \frac{2\omega^{6}+p\omega^{4}-r}{\omega ^{2}[(b_{0}-b_{2}\omega^{2})^{2}+(b_{1}\omega)^{2}]} \biggr\} . \end{aligned}$$

Here, $p>0$ and $r>0$ under condition (4.9). Thus $\frac{d(\operatorname{Re}\lambda)}{d\tau} \vert _{\tau=\tau_{0}}>0$. This result shows that the root of characteristic (4.2) crosses the imaginary axis from left to right as *τ* continuously varies from a number less than $\tau_{0}$ to greater than $\tau_{0}$. Therefore, the conditions for Hopf bifurcation [33] are satisfied at $\tau=\tau_{0}$. From Theorem 4.2 and our analysis we obtain the following theorem.

### Theorem 4.3

*Suppose that* $\mathcal{R}_{0}>1$. *Then the endemic equilibrium* $\mathcal{E}^{*}$ *of model* (2.1) *is*

(i) *absolutely stable for* $\tau\geq0$ *whenever* $\frac{k_{1} k_{2}}{\beta_{E} k_{2} + \beta_{I} \sigma}<\beta< \frac{2 k_{1} k_{2} -\sigma\alpha}{\beta_{E} k_{2} + \beta_{I} \sigma}$ *and*
(ii) *conditionally stable for* $\tau\in[0,\tau_{0})$ *whenever* $\beta> \frac{3k_{1} k_{2}-\sigma \alpha}{\beta_{E}k_{2}+\beta_{I}\sigma}$. *System* (2.1) *with* $\tau=\tau_{0}$ *given in* (4.10) *undergoes a Hopf bifurcation*.

## Direction and stability of the Hopf bifurcation

In Sect. 4, we obtained conditions under which the periodic solutions bifurcate from endemic equilibrium $\mathcal{E}^{*}$ at the critical values $\tau_{n}$ via the Hopf bifurcation. However, Theorems 4.3(ii) cannot determine the stability and direction of bifurcating periodic solutions, that is, the periodic solutions may exist for $\tau>\tau_{0}$ near $\tau_{0}$. In this section, the direction, stability, and periods of these periodic solutions are determined by using the normal theory and the center manifold theorem [34].

Let $u_{1}(t)=S(t)-S^{*}, u_{2}(t)=E(t)-E^{*}, u_{3}(t)=I(t)-I^{*}, u_{4}(t)=R(t)-R^{*}, x_{i}(t)=u_{i}(\tau t), \tau=\tau_{0}+\mu$, where $\tau_{0}$ is defined by (4.10), and $\mu\in\mathbb{R}$. System (2.1) can be written as a functional differential equation in $\mathbb{C}=\mathbb{C}([-1,0],\mathbb{R}^{4})$ as

5.1 $$\begin{aligned} x' = L_{\mu}(x_{t})+f( \mu,x_{t}), \end{aligned}$$

where $x(t)= (x_{1}(t),x_{2}(t),x_{3}(t),x_{4}(t) )^{T}\in\mathbb{R}^{4}$, and $L_{\mu}:\mathbb{C}\rightarrow\mathbb{R}^{4}$ and $f:\mathbb{R}\times\mathbb{C}\rightarrow\mathbb{R}^{4}$ are given by

5.2 $$\begin{array}{rl} L_{\mu }(\phi )= & (\tau_{n}+\mu )\left[\begin{array}{cccc} -\mu & 0 & 0 & 0\\ 0 & -k_{1} & 0 & 0\\ 0 & \sigma & -k_{2} & 0\\ 0 & \kappa & \gamma & -\mu \end{array}\right]\left[\begin{array}{c} \phi_{1}(0)\\ \phi_{2}(0)\\ \phi_{3}(0)\\ \phi_{4}(0) \end{array}\right]\\ & +(\tau_{n}+\mu )\left[\begin{array}{cccc} -m_{1} & -m_{2} & -m_{3} & -m_{4}\\ m_{1} & m_{2} & m_{3} & m_{4}\\ 0 & 0 & 0 & 0\\ 0 & 0 & 0 & 0 \end{array}\right]\left[\begin{array}{c} \phi_{1}(-1)\\ \phi_{2}(-1)\\ \phi_{3}(-1)\\ \phi_{4}(-1) \end{array}\right] \end{array}$$

and

5.3 $$f(\mu ,\phi )=(\tau_{n}+\mu )\left[\begin{array}{c} F_{1}\\ -F_{1}\\ 0\\ 0 \end{array}\right],$$

where

$$\begin{aligned} F_{1} ={}& l_{1}\phi_{1}^{2}(-1)+l_{2} \phi_{2}^{2}(-1)+l_{3}\phi_{3}^{2}(-1)+l_{4} \phi _{4}^{2}(-1)+l_{5}\phi_{1}(-1) \phi_{2}(-1)+l_{6}\phi_{1}(-1)\phi_{3}(-1) \\ &{}+l_{7}\phi_{1}(-1)\phi_{4}(-1)+l_{8} \phi_{2}(-1)\phi_{3}(-1)+l_{9}\phi _{2}(-1)\phi_{4}(-1)+l_{10}\phi_{3}(-1) \phi_{4}(-1) \end{aligned}$$

with

$$\begin{aligned} &l_{1}=\frac{Q^{*}}{N^{*}} \biggl(1-\frac{1}{R_{0}} \biggr),\qquad l_{2}=\frac {\beta_{1}-Q^{*}}{R_{0}N^{*}},\qquad l_{3}=\frac{\beta_{2}-Q^{*}}{R_{0}N^{*}},\qquad l_{4}=-\frac{Q^{*}}{R_{0}N^{*}}, \\ &l_{5}=\frac{Q^{*}}{N^{*}} \biggl(1-\frac{2}{R_{0}} \biggr)- \frac{\beta _{1}}{N^{*}} \biggl(1-\frac{1}{R_{0}} \biggr),\qquad l_{6}= \frac{Q^{*}}{N^{*}} \biggl(1-\frac{2}{R_{0}} \biggr)-\frac{\beta _{2}}{N^{*}} \biggl(1-\frac{1}{R_{0}} \biggr), \\ &l_{7}=\frac{Q^{*}}{N^{*}} \biggl(1-\frac{2}{R_{0}} \biggr),\qquad l_{8}=\frac{\beta_{1}+\beta_{2}-2Q^{*}}{R_{0}N^{*}}, \qquad l_{9}=\frac{\beta_{1}-2Q^{*}}{R_{0}N^{*}},\qquad l_{10} = \frac{\beta_{2}-2Q^{*}}{R_{0}N^{*}}, \\ &m_{1}=Q^{*} \biggl(1-\frac{1}{R_{0}} \biggr),\qquad m_{2}= \frac{\beta_{1}-Q^{*}}{R_{0}},\qquad m_{3}=\frac{\beta _{2}-Q^{*}}{R_{0}} \quad\text{and}\quad m_{4}=-\frac{Q^{*}}{R_{0}}. \end{aligned}$$

By the Riezs representation theorem there exists a function $\eta (\theta,\mu)$ of bounded variation for $\theta\in[-1,0]$ such that

5.4 $$ L_{\mu}(\phi)= \int_{-1}^{0}\,d\eta(\theta,\mu)\phi(\theta) \quad\text{for } \phi\in\mathbb{C}. $$

In fact, if we choose

5.5 $$\begin{array}{rl} \eta (\theta ,\mu )= & \left(\tau_{n}+\mu )\left[\begin{array}{cccc} -\mu & 0 & 0 & 0\\ 0 & -k_{1} & 0 & 0\\ 0 & \sigma & -k_{2} & 0\\ 0 & \kappa & \gamma & -\mu \end{array}\right]\delta (\theta \right)\\ & -(\tau_{n}+\mu )\left[\begin{array}{cccc} -m_{1} & -m_{2} & -m_{3} & -m_{4}\\ m_{1} & m_{2} & m_{3} & m_{4}\\ 0 & 0 & 0 & 0\\ 0 & 0 & 0 & 0 \end{array}\right]\delta (\theta +1), \end{array}$$

where $\delta(\theta)$ is Dirac delta function, then (5.4) is satisfied.

For $\phi\in\mathbb{C}^{1}([-1,0],\mathbb{R}^{4})$, define

$$\begin{aligned} A(\mu)\phi= \textstyle\begin{cases} \frac{d\phi(\theta)}{d\theta}, & \theta\in[-1,0),\\ \int_{-1}^{0}\,d\eta(s,\mu)\phi(s), & \theta=0, \end{cases}\displaystyle \end{aligned}$$

and

$$\begin{aligned} R(\mu)\phi= \textstyle\begin{cases} 0, & \theta\in[-1,0),\\ f(\mu,\phi), & \theta=0. \end{cases}\displaystyle \end{aligned}$$

Then system (5.1) is equivalent to

5.6 $$ x'_{t}=A(\mu)x_{t}+R( \mu)x_{t}, $$

where $x_{t}(\theta)=x(t+\theta)$ for $\theta\in[-1,0]$.

For $\psi\in\mathbb{C}^{1}([0,1],(\mathbb{R}^{4})^{*})$, define

$$\begin{aligned} A^{*}\psi(s)= \textstyle\begin{cases} -\frac{d\psi(s)}{ds}, & s\in(0,1],\\ \int_{-1}^{0} \,d\eta^{T}(t,0)\psi(-t), & s=0, \end{cases}\displaystyle \end{aligned}$$

and the bilinear inner product

5.7 $$ \bigl\langle \psi(s),\phi(\theta)\bigr\rangle =\overline{\psi}(0) \phi (0)- \int_{\theta=-1}^{0} \int_{\xi=0}^{\theta}\overline{\psi}(\xi -\theta)\,d\eta( \theta)\phi(\xi)\,d\xi, $$

where $\eta(\theta)=\eta(\theta,0)$. Then $A(0)$ and $A^{*}$ are adjoint operators. We know that $\pm \text{i}\omega_{0}\tau_{n}$ are eigenvalues of $A(0)$, as discussed in Sect. 3. Thus they are also eigenvalues of $A^{*}$. We need to compute the eigenvectors of $A(0)$ and $A^{*}$ corresponding to the eigenvalues $\text{i}\omega_{0}\tau_{n}$ and $-\text{i}\omega_{0}\tau_{n}$, respectively.

Suppose $v(\theta) = (1,v_{1},v_{2},v_{3})^{T}e^{\text{i}\omega_{0}\tau_{n}\theta}$ is the eigenvector of $A(0)$ corresponding to $\text{i}\omega_{0}\tau_{n}$. Then $A(0)v(0)=\text{i}\omega_{0}\tau_{n} v(0)$. It follows from the definition of $A(0)$ and (5.2)–(5.5) that

$$\tau_{n}\left[\begin{array}{cccc} -\mu & 0 & 0 & 0\\ 0 & -k_{1} & 0 & 0\\ 0 & \sigma & -k_{2} & 0\\ 0 & \kappa & \gamma & -\mu \end{array}\right]v(0)+\tau_{n}\left[\begin{array}{cccc} -m_{1} & -m_{2} & -m_{3} & -m_{4}\\ m_{1} & m_{2} & m_{3} & m_{4}\\ 0 & 0 & 0 & 0\\ 0 & 0 & 0 & 0 \end{array}\right]v(-1)=\text{i}\omega_{0}\tau_{n}v(0).$$

Then, for $v(-1)=v(0)e^{-\text{i}\omega_{0}\tau_{n}}$, we obtain

$$ v_{1}=-\frac{\mu+\text{i}\omega_{0}}{k_{1}+\text{i}\omega_{0}}, \qquad v_{2}=- \frac{\sigma(\mu+\text{i}\omega_{0})}{(k_{1}+\text{i}\omega _{0})(k_{2}+\text{i}\omega_{0})},\qquad v_{3}=-\frac{\kappa(k_{2}+\text{i}\omega_{0})+\sigma\gamma}{(k_{1}+\text{i}\omega_{0})(k_{2}+\text{i}\omega_{0})}. $$

Similarly, we can obtain the eigenvector $v^{*}(s)=D(1,v^{*}_{1},v^{*}_{2},v^{*}_{3})e^{\text{i}\omega_{0}\tau_{n} s}$ of $A^{*}$ corresponding to $-\text{i}\omega_{0}\tau_{n}$, where

$$\begin{aligned} v^{*}_{1}=\frac{\mu-\text{i}\omega_{0}+m_{1}e^{\text{i}w_{0}\tau_{n}}}{m_{1}e^{\text{i}\omega_{0}\tau_{n}}}, \qquad v^{*}_{2}=\frac{m_{3}(\text{i}\omega_{0}-\mu)-m_{4}\gamma}{m_{1}(\text{i}\omega_{0}-k_{2})},\qquad v^{*}_{3}=\frac{m_{4}}{m_{1}}. \end{aligned}$$

To ensure that $\langle v^{*}(s),v(\theta)\rangle=1$, we have to determine the value of *D*. By (5.7) we have

$$\begin{aligned} &\bigl\langle v^{*}(s),v(\theta)\bigr\rangle \\ &\quad= \overline{D}\bigl(1, \bar{v}^{*}_{1}, \bar {v}^{*}_{2}, \bar{v}^{*}_{3}\bigr) (1, v_{1}, v_{2}, v_{3})^{T} \\ &\qquad{}-\int_{\theta=-1}^{0}\int_{\xi=0}^{\theta}\overline{D}\bigl(1, \bar{v}^{*}_{1}, \bar{v}^{*}_{2},\bar{v}^{*}_{3} \bigr)e^{-\text{i}\omega_{0}\tau_{n}(\xi -\theta)}\,d\eta(\theta) (1, v_{1}, v_{2}, v_{3})^{T} e^{\text{i}\omega_{0}\tau _{n}\xi}\,d\xi \\ &\quad= \overline{D} \biggl\{ 1+v_{1}\bar{v}^{*}_{1}+v_{2} \bar{v}^{*}_{2}+v_{3}\bar {v}^{*}_{3}-\int_{\theta=-1}^{0}\bigl(1, \bar{v}^{*}_{1}, \bar{v}^{*}_{2}, \bar {v}^{*}_{3}\bigr)\theta e^{\text{i}\omega_{0}\tau_{n}\theta}\,d \eta(\theta) (1, v_{1}, v_{2}, v_{3})^{T} \biggr\} \\ &\quad = \overline{D} \bigl\{ 1+v_{1}\bar{v}^{*}_{1}+v_{2} \bar{v}^{*}_{2}+v_{3}\bar {v}^{*}_{3}+ \tau_{n}\bigl(\bar{v}^{*}_{1}-1\bigr) (m_{1}+m_{2}v_{1}+m_{3}v_{2}+m_{4}v_{3})e^{-\text{i}\omega_{0}\tau_{n}} \bigr\} . \end{aligned}$$

Therefore we can choose *D* as

$$\begin{aligned} \overline{D}=\frac{1}{1+v_{1}\bar{v}^{*}_{1}+v_{2}\bar{v}^{*}_{2}+v_{3}\bar {v}^{*}_{3}+\tau_{n}(\bar{v}^{*}_{1}-1) (m_{1}+m_{2}v_{1}+m_{3}v_{2}+m_{4}v_{3})e^{-\text{i}\omega_{0}\tau_{n}}}. \end{aligned}$$

Using the same notation as in [34], we will compute the coordinates describing the center manifold $C_{0}$ at $\mu=0$. Define

5.8 $$ \left.\begin{aligned} &z(t)=\bigl\langle v^{*},x_{t}\bigr\rangle , \\ &W(t,\theta)=x_{t}-z(t)v(\theta)-\bar{z}(t)\bar{v}( \theta)=x_{t}-2\operatorname {Re}\bigl\{ z(t)v(\theta)\bigr\} . \end{aligned} \right\} $$

On the center manifold $C_{0}$, we have

5.9 $$\begin{aligned} W(t,\theta)& = W\bigl(z(t),\bar{z}(t),\theta\bigr) \\ & = W_{20}(\theta)\frac{z^{2}}{2}+W_{11}(\theta)z\bar {z}+W_{02}(\theta)\frac{\bar{z}^{2}}{2}+\cdots, \end{aligned}$$

where *z* and *z̄* are local coordinates for the center manifold $C_{0}$ in the direction of $v^{*}$ and $\bar{v}^{*}$. Note that *W* is real if $x_{t}$ is real. Here we consider only real solutions. For the solution $x_{t}\in C_{0}$ of (5.6), since $\mu=0$, we have

$$\begin{aligned} z'(t) &= \text{i}\omega_{0} \tau_{n} z(t)+\bar{v}^{*}(0)f\bigl(0,W\bigl(z(t),\bar {z}(t),0\bigr)\bigr)+2\operatorname{Re}\bigl\{ z(t),v(\theta)\bigr\} \\ &\triangleq \text{i}\omega_{0} \tau_{n} z(t)+g(z,\bar{z}), \end{aligned}$$

where

5.10 $$\begin{aligned} g(z,\bar{z})=\bar{v}^{*}(0)f_{0}(z,\bar{z})=g_{20} \frac{z^{2}}{2}+g_{11}z\bar{z}+g_{02}\frac{\bar {z}^{2}}{2}+g_{21}\frac{z^{2}\bar{z}}{2}+\cdots. \end{aligned}$$

From (5.8) and (5.9) we have $x_{t}=W(z,\bar{z},\theta)+zv+\bar{z}\bar{v}$. Thus,

$$x_{t}=\left[\begin{array}{c} x_{1t}(\theta )\\ x_{2t}(\theta )\\ x_{3t}(\theta )\\ x_{4t}(\theta ) \end{array}\right]=\left[\begin{array}{c} W^{\left(1\right)}(z,\bar{z},\theta )\\ W^{\left(2\right)}(z,\bar{z},\theta )\\ W^{\left(3\right)}(z,\bar{z},\theta )\\ W^{\left(4\right)}(z,\bar{z},\theta ) \end{array}\right]+z\left[\begin{array}{c} 1\\ v_{1}\\ v_{2}\\ v_{3} \end{array}\right]e^{\text{i}\omega_{0}\tau_{n}\theta }+\bar{z}\left[\begin{array}{c} 1\\ {\bar{v}}_{1}\\ {\bar{v}}_{2}\\ {\bar{v}}_{3} \end{array}\right]e^{-\text{i}\omega_{0}\tau_{n}\theta },$$

and

$$\begin{aligned} &x_{1t}(-1) = ze^{-\text{i}\omega_{0}\tau_{n}}+\bar{z}e^{\text{i}\omega_{0}\tau _{n}}+W_{20}^{(1)}(-1) \frac{z^{2}}{2}+W_{11}^{(1)}(-1)z\bar {z}+W_{02}^{(1)}(-1)\frac{\bar{z}^{2}}{2}+\cdots, \\ &x_{2t}(-1) = zv_{1}e^{-\text{i}\omega_{0}\tau_{n}}+\bar{z} \bar{v}_{1}e^{\text{i}\omega_{0}\tau_{n}}+W_{20}^{(2)}(-1)\frac {z^{2}}{2}+W_{11}^{(2)}(-1)z\bar{z}+W_{02}^{(2)}(-1) \frac{\bar {z}^{2}}{2}+\cdots, \\ &x_{3t}(-1) = zv_{2}e^{-\text{i}\omega_{0}\tau_{n}}+\bar{z} \bar{v}_{2}e^{\text{i}\omega_{0}\tau_{n}}+W_{20}^{(3)}(-1)\frac {z^{2}}{2}+W_{11}^{(3)}(-1)z\bar{z}+W_{02}^{(3)}(-1) \frac{\bar {z}^{2}}{2}+\cdots, \\ &x_{4t}(-1) = zv_{3}e^{-\text{i}\omega_{0}\tau_{n}}+\bar{z} \bar{v}_{3}e^{\text{i}\omega_{0}\tau_{n}}+W_{20}^{(4)}(-1)\frac {z^{2}}{2}+W_{11}^{(4)}(-1)z\bar{z}+W_{02}^{(4)}(-1) \frac{\bar {z}^{2}}{2}+\cdots. \end{aligned}$$

From this and from (5.3) it follows that

$$\begin{array}{rl} g(z,\bar{z}) & ={\bar{v}}^{*}(0)f_{0}(z,\bar{z})\\ & ={\bar{v}}^{*}(0)f(0,x_{t})\\ & =\bar{D}\left(1,{\bar{v}}_{1}^{*},{\bar{v}}_{2}^{*},{\bar{v}}_{3}^{*}\right)\tau_{n}\left[\begin{array}{c} G\\ -G\\ 0\\ 0 \end{array}\right]\\ & =\bar{D}\left(1-{\bar{v}}_{1}^{*}\right)\tau_{n}G, \end{array}$$

where

$$\begin{aligned} G ={}& l_{1} x_{1t}^{2}(-1)+l_{2} x_{2t}^{2}(-1)+l_{3} x_{3t}^{2}(-1)+l_{4} x_{4t}^{2}(-1)+l_{5} x_{1t}(-1)x_{2t}(-1) \\ &{}+l_{6} x_{1t}(-1)x_{3t}(-1)+l_{7} x_{1t}(-1)x_{4t}(-1)+l_{8} x_{2t}(-1)x_{3t}(-1) +l_{9} x_{2t}(-1)x_{4t}(-1) \\ &{}+l_{10} x_{3t}(-1)x_{4t}(-1). \end{aligned}$$

Comparing the coefficients with (5.10), we have

5.11 $$\begin{aligned} g_{20} ={}& 2\overline{D} \tau_{n}\bigl(1-\bar{v}_{1}^{*}\bigr)e^{-2\text{i}\omega_{0}\tau _{n}} \bigl\{ l_{1}+l_{2}v_{1}^{2}+l_{3}v_{2}^{2}+l_{4}v_{3}^{2}+l_{5}v_{1} +l_{6}v_{2}+l_{7}v_{3}+l_{8}v_{1}v_{2} \\ &{}+l_{9}v_{1}v_{3}+l_{10}v_{2}v_{3} \bigr\} , \end{aligned}$$

5.12 $$\begin{aligned} g_{02} ={}& 2\overline{D}\tau_{n}\bigl(1-\bar{v}_{1}^{*} \bigr)e^{2\text{i}\omega_{0}\tau _{n}} \bigl\{ l_{1}+l_{2}\bar{v}_{1}^{2}+l_{3} \bar{v}_{2}^{2}+l_{4}\bar{v}_{3}^{2}+l_{5} \bar{v}_{1} +l_{6}\bar{v}_{2}+l_{7} \bar{v}_{3}+l_{8}\bar{v}_{1}\bar{v}_{2} \\ &{}+l_{9}\bar{v}_{1}\bar{v}_{3}+l_{10} \bar{v}_{2}\bar{v}_{3} \bigr\} , \end{aligned}$$

5.13 $$\begin{aligned} g_{11} ={}& \overline{D}\tau_{n}\bigl(1-\bar{v}_{1}^{*} \bigr) \bigl\{ 2l_{1}+2l_{2}v_{1}\bar {v}_{1}+2l_{3}v_{2}\bar{v}_{2}+2l_{4}v_{3} \bar{v}_{3} +l_{5}(v_{1}+\bar{v}_{1})+l_{6}(v_{2}+ \bar{v}_{2}) \\ &{}+l_{7}(v_{3}+\bar{v}_{3})+l_{8}(v_{1} \bar{v}_{2}+\bar{v}_{1}v_{2}) +l_{9}(v_{1} \bar{v}_{3}+\bar{v}_{1}v_{3})+l_{10}(v_{2} \bar{v}_{3}+\bar {v}_{2}v_{3}) \bigr\} , \end{aligned}$$

5.14 $$\begin{aligned} g_{21} ={}& 2\overline{D}\tau_{n}\bigl(1- \bar{v}_{1}^{*}\bigr) \biggl\{ 2l_{1}W_{11}^{(1)}(-1)e^{-\text{i}\omega_{0}\tau_{n}} +l_{1}W_{20}^{(1)}(-1)e^{\text{i}\omega_{0}\tau _{n}}+2l_{2}v_{1}W_{11}^{(2)}(-1)e^{-\text{i}\omega_{0}\tau_{n}} \\ &{}+l_{2}\bar{v}_{1}W_{20}^{(2)}(-1)e^{\text{i}\omega_{0}\tau_{n}} +2l_{3}v_{2}W_{11}^{(3)}(-1)e^{-\text{i}\omega_{0}\tau_{n}}+l_{3} \bar {v}_{2}W_{20}^{(3)}(-1)e^{\text{i}\omega_{0}\tau_{n}} \\ &{}+2l_{4}v_{3}W_{11}^{(4)}(-1)e^{-\text{i}\omega_{0}\tau_{n}}+l_{4} \bar {v}_{3}W_{20}^{(4)}(-1)e^{\text{i}\omega_{0}\tau _{n}}+l_{5}W_{11}^{(2)}(-1)e^{-\text{i}\omega_{0}\tau_{n}} \\ &{}+\frac{l_{5}}{2}W_{20}^{(2)}(-1)e^{\text{i}\omega_{0}\tau _{n}}+l_{5}v_{1}W_{11}^{(1)}(-1)e^{-\text{i}\omega_{0}\tau_{n}}+l_{5} \frac{\bar {v}_{1}}{2}W_{20}^{(1)}(-1)e^{\text{i}\omega_{0}\tau_{n}} \\ &{}+l_{6}W_{11}^{(3)}(-1)e^{-\text{i}\omega_{0}\tau_{n}}+\frac {l_{6}}{2}W_{20}^{(3)}(-1)e^{\text{i}\omega_{0}\tau _{n}}+l_{6}v_{2}W_{11}^{(1)}(-1)e^{-\text{i}\omega_{0}\tau_{n}} \\ &{}+l_{6}\frac{\bar{v}_{2}}{2}W_{20}^{(1)}(-1)e^{\text{i}\omega _{0}\tau_{n}}+l_{7}W_{11}^{(4)}(-1)e^{-\text{i}\omega_{0}\tau_{n}}+ \frac {l_{7}}{2}W_{20}^{(4)}(-1)e^{\text{i}\omega_{0}\tau_{n}} \\ &{}+l_{7}v_{3}W_{11}^{(1)}(-1)e^{-\text{i}\omega_{0}\tau_{n}}+l_{7} \frac {\bar{v}_{3}}{2}W_{20}^{(1)}(-1)e^{\text{i}\omega_{0}\tau _{n}}+l_{8}v_{1}W_{11}^{(3)}(-1)e^{-\text{i}\omega_{0}\tau_{n}} \\ &{}+l_{8}\frac{\bar{v}_{1}}{2}W_{20}^{(3)}(-1)e^{\text{i}\omega _{0}\tau_{n}}+l_{8}v_{2}W_{11}^{(2)}(-1)e^{-\text{i}\omega_{0}\tau_{n}}+l_{8} \frac {\bar{v}_{2}}{2}W_{20}^{(2)}(-1)e^{\text{i}\omega_{0}\tau_{n}} \\ &{}+l_{9}v_{1}W_{11}^{(4)}(-1)e^{-\text{i}\omega_{0}\tau_{n}}+l_{9} \frac {\bar{v}_{1}}{2}W_{20}^{(4)}(-1)e^{\text{i}\omega_{0}\tau _{n}}+l_{9}v_{3}W_{11}^{(2)}(-1)e^{-\text{i}\omega_{0}\tau_{n}} \\ &{}+l_{9}\frac{\bar{v}_{3}}{2}W_{20}^{(2)}(-1)e^{\text{i}\omega _{0}\tau_{n}}+l_{10}v_{2}W_{11}^{(4)}(-1)e^{-\text{i}\omega_{0}\tau_{n}}+l_{10} \frac{\bar{v}_{2}}{2}W_{20}^{(4)}(-1)e^{\text{i}\omega_{0}\tau_{n}} \\ &{}+l_{10}v_{3}W_{11}^{(3)}(-1)e^{-\text{i}\omega_{0}\tau_{n}}+l_{10} \frac{\bar{v}_{3}}{2}W_{20}^{(3)}(-1)e^{\text{i}\omega_{0}\tau_{n}} \biggr\} . \end{aligned}$$

Since $W_{20}(\theta)$ and $W_{11}(\theta)$ are in $g_{21}$, we need to compute them. From (5.6) and (5.8) we have

5.15 $$\begin{aligned} \dot{W} &= \dot{x}_{t}-\dot{z}v-\dot{\bar{z}}\bar{v} \\ &= \textstyle\begin{cases} A(0)W-2\operatorname{Re} \{\bar{v}^{*}(0)f_{0}(z,\bar{z})v(\theta) \} , & \theta\in[-1,0), \\ A(0)W-2\operatorname{Re} \{\bar{v}^{*}(0)f_{0}(z,\bar{z})v(0) \} +f_{0}(z,\bar{z}), & \theta=0, \end{cases}\displaystyle \\ &\triangleq A(0)W+H(z,\bar{z},\theta), \end{aligned}$$

where

5.16 $$ H(z,\bar{z},\theta)=H_{20}(\theta)\frac{z^{2}}{2}+H_{11}(\theta )z\bar{z}+H_{02}(\theta)\frac{\bar{z}^{2}}{2}+\cdots. $$

Substituting the series (5.9) and (5.16) into (5.15) and comparing the coefficients, we have

5.17 $$ \bigl(A(0)-2\text{i}\omega_{0}\tau_{n}\mathbf{I} \bigr)W_{20}(\theta)=-H_{20}(\theta ),\qquad A(0)W_{11}( \theta)=-H_{11}(\theta), $$

where **I** is the identity matrix. By (5.15) we know that, for $\theta\in[-1,0)$,

$$\begin{aligned} H(z,\bar{z},\theta)={}&{ -}\bar{v}^{*}(0)f_{0}(z,\bar{z})v(\theta )-v^{*}(0) \bar{f}_{0}(z,\bar{z})\bar{v}(\theta) \\ ={}& {-}g(z,\bar{z})v(\theta)-\bar{g}(z,\bar{z})\bar{v}(\theta ) \\ ={}&{ -} \biggl(g_{20}\frac{z^{2}}{2}+g_{11}z \bar{z}+g_{02}\frac {\bar{z}^{2}}{2}+g_{21}\frac{z^{2}\bar{z}}{2}+\cdots \biggr)v(\theta) \\ &{}- \biggl(\bar{g}_{20}\frac{\bar{z}^{2}}{2}+\bar{g}_{11}z \bar {z}+\bar{g}_{02}\frac{z^{2}}{2}+\bar{g}_{21}\frac{\bar {z}^{2}z}{2}+\cdots \biggr)\bar{v}(\theta). \end{aligned}$$

Comparing the coefficients with (5.16), we obtain

5.18 $$ \left.\begin{aligned} H_{20}( \theta) = -g_{20}v(\theta)-\bar{g}_{02}\bar{v}(\theta), \\ H_{11}(\theta) = -g_{11}v(\theta)-\bar{g}_{11} \bar{v}(\theta). \end{aligned} \right \} $$

From (5.17)–(5.18) and the definition of $A(0)$ we have

5.19 $$ \left. \begin{aligned} W'_{20}( \theta)=2\text{i}\omega_{0}\tau_{n} W_{20}( \theta)+g_{20}v(\theta )+\bar{g}_{02}\bar{v}(\theta), \\ W'_{11}(\theta)=g_{11}v(\theta)- \bar{g}_{11}\bar{v}(\theta). \end{aligned} \right \} $$

Noticing that $v(\theta)=v(0)e^{\text{i}\omega_{0}\tau_{n}\theta}$ and solving system (5.19), we obtain

5.20 $$\begin{aligned} &W_{20}(\theta) = \frac{\text{i}g_{20}}{\omega_{0}\tau_{n}}v(0)e^{\text{i}\omega_{0}\tau_{n}\theta} +\frac{\text{i}\bar{g}_{02}}{3\omega_{0}\tau_{n}} \bar{v}(0)e^{-\text{i}\omega_{0}\tau_{n}\theta} +E_{1}e^{2\text{i}\omega_{0}\tau_{n}\theta}, \end{aligned}$$

5.21 $$\begin{aligned} &W_{11}(\theta) = -\frac{\text{i}g_{11}}{\omega_{0}\tau_{n}}v(0)e^{\text{i}\omega_{0}\tau_{n}\theta} +\frac{\text{i}\bar{g}_{11}}{\omega_{0}\tau_{n}}\bar{v}(0)e^{-\text{i}\omega_{0}\tau_{n}\theta}+E_{2}, \end{aligned}$$

where $E_{i}=(E_{i}^{(1)}, E_{i}^{(2)}, E_{i}^{(3)}, E_{i}^{(4)})^{T}, i=1,2, \in\mathbb{R}^{4}$, are constant vectors.

We will further find the values of $E_{1}$ and $E_{2}$. From the definition of $A(0)$ and (5.17) we have

5.22 $$ \int_{-1}^{0}\,d\eta(\theta)W_{20}( \theta)=2\text{i}\omega_{0}\tau _{n}W_{20}(0)-H_{20}(0) $$

and

5.23 $$ \int_{-1}^{0}\,d\eta(\theta)W_{11}( \theta)=-H_{11}(0), $$

where $\eta(\theta)=\eta(0,\theta)$. By (5.15) we know that, when $\theta=0$,

$$\begin{aligned} H(z,\bar{z},0)& = -2\operatorname{Re} \bigl\{ \bar{v}^{*}(0)f_{0}(z, \bar {z})v(0) \bigr\} +f_{0}(z,\bar{z}) \\ & = -\bar{v}^{*}(0)f_{0}(z,\bar{z})v(0)-v^{*}(0)\bar{f}_{0}(z, \bar{z})\bar {v}(0)+f_{0}(z,\bar{z}) \\ & = -g(z,\bar{z})v(0)-\bar{g}(z,\bar{z})\bar{v}(0)+f_{0}(z,\bar{z}). \end{aligned}$$

In view of (5.16), this gives

5.24 $$H_{20}(0)=-g_{20}v(0)-{\bar{g}}_{02}\bar{v}(0)+2\tau_{n}\left[\begin{array}{c} G_{2}\\ -G_{2}\\ 0\\ 0 \end{array}\right],$$

5.25 $$H_{11}(0)=-g_{11}v(0)-{\bar{g}}_{11}\bar{v}(0)+2\tau_{n}\left[\begin{array}{c} G_{3}\\ -G_{3}\\ 0\\ 0 \end{array}\right],$$

where

$$\begin{aligned} G_{2} ={}& e^{-2\text{i}\omega_{0}\tau_{n}} \bigl\{ l_{1}+l_{2}v_{1}^{2}+l_{3}v_{2}^{2}+l_{4}v_{3}^{2}+l_{5}v_{1}+l_{6}v_{2}+l_{7}v_{3}+l_{8}v_{1}v_{2}+l_{9}v_{1}v_{3}+l_{10}v_{2}v_{3} \bigr\} , \\ G_{3} ={}& l_{1}+l_{2}v_{1} \bar{v}_{1}+l_{3}v_{2}\bar{v}_{2}+l_{4}v_{3} \bar{v}_{3}+l_{5}\operatorname {Re}\{v_{1}\} +l_{6}\operatorname{Re}\{v_{2}\}+l_{7}\operatorname{Re} \{v_{3}\}+l_{8}\operatorname{Re}\{v_{1}\bar {v}_{2}\} \\ &{}+l_{9}\operatorname{Re}\{v_{1}\bar{v}_{3} \}+l_{10}\operatorname{Re}\{v_{2}\bar{v}_{3}\}. \end{aligned}$$

Since $\text{i}\omega_{0}\tau_{n}$ is an eigenvalue of $A(0)$ and $v(0)$ is the corresponding eigenvector, we obtain

$$\begin{aligned} & \biggl(\text{i}\omega_{0} \tau_{n} I-\int_{-1}^{0}e^{\text{i}\theta\omega _{0}\tau_{n}}\,d\eta(\theta) \biggr)v(0) = 0, \\ &\biggl(-\text{i}\omega_{0}\tau_{n} I-\int_{-1}^{0}e^{-\text{i}\theta\omega _{0}\tau_{n}}\,d\eta(\theta) \biggr)v(0) = 0. \end{aligned}$$

Substituting (5.20) and (5.24) into (5.22) yields

$$\left(2\text{i}\omega_{0}\tau_{n}I-\int_{-1}^{0}e^{2\text{i}\omega_{0}\tau_{n}\theta }\;d\eta (\theta )\right)E_{1}=2\tau_{n}\left[\begin{array}{c} G_{2}\\ -G_{2}\\ 0\\ 0 \end{array}\right],$$

which leads to

$$\begin{array}{rl} & \left[\begin{array}{cccc} 2\text{i}\omega_{0}+\mu +m_{1}e^{-2\text{i}\omega_{0}\tau_{n}} & m_{2}e^{-2\text{i}\omega_{0}\tau_{n}} & m_{3}e^{-2\text{i}\omega_{0}\tau_{n}} & m_{4}e^{-2\text{i}\omega_{0}\tau_{n}}\\ -m_{1}e^{-2\text{i}\omega_{0}\tau_{n}} & 2\text{i}\omega_{0}+k_{1}-m_{2}e^{-2\text{i}\omega_{0}\tau_{n}} & -m_{3}e^{-2\text{i}\omega_{0}\tau_{n}} & -m_{4}e^{-2\text{i}\omega_{0}\tau_{n}}\\ 0 & -\sigma & 2\text{i}\omega_{0}+k_{2} & 0\\ 0 & -\kappa & -\gamma & 2\text{i}\omega_{0}+\mu \end{array}\right]\left[\begin{array}{c} E_{1}^{\left(1\right)}\\ E_{1}^{\left(2\right)}\\ E_{1}^{\left(3\right)}\\ E_{1}^{\left(4\right)} \end{array}\right]\\ & \;=2\left[\begin{array}{c} G_{2}\\ -G_{2}\\ 0\\ 0 \end{array}\right]. \end{array}$$

Then it follows that

$$\begin{aligned} &E_{1}^{(1)} = \frac {2h_{1}h_{2}G_{2}}{h_{1}h_{2}h_{3}+(h_{1}h_{2}m_{1}-h_{2}h_{3}m_{2}-\sigma h_{3}m_{3}-(\kappa h_{2}+\gamma\sigma)m_{4})e^{-2\text{i}\omega_{0}\tau_{n}}}, \\ &E_{1}^{(2)} = \frac {-2h_{2}h_{3}G_{2}}{h_{1}h_{2}h_{3}+(h_{1}h_{2}m_{1}-h_{2}h_{3}m_{2}-\sigma h_{3}m_{3}-(\kappa h_{2}+\gamma\sigma)m_{4})e^{-2\text{i}\omega_{0}\tau_{n}}}, \\ &E_{1}^{(3)} = \frac{-2\sigma h_{3}G_{2}}{h_{1}h_{2}h_{3}+(h_{1}h_{2}m_{1}-h_{2}h_{3}m_{2}-\sigma h_{3}m_{3}-(\kappa h_{2}+\gamma \sigma)m_{4})e^{-2\text{i}\omega_{0}\tau_{n}}}, \\ &E_{1}^{(4)} = \frac{-2(\kappa h_{2}+\sigma\gamma )G_{2}}{h_{1}h_{2}h_{3}+(h_{1}h_{2}m_{1}-h_{2}h_{3}m_{2}-\sigma h_{3}m_{3}-(\kappa h_{2}+\gamma \sigma)m_{4})e^{-2\text{i}\omega_{0}\tau_{n}}}, \end{aligned}$$

where $h_{1}=2\text{i}\omega_{0}+k_{1}, h_{2}=2\text{i}\omega_{0}+k_{2}$, and $h_{3}=2\text{i}\omega_{0}+\mu$.

Similarly, substituting (5.21) and (5.25) into (5.23), we obtain

$$\left[\begin{array}{cccc} -\mu -m_{1} & -m_{2} & -m_{3} & -m_{4}\\ m_{1} & -k_{1}+m_{2} & m_{3} & m_{4}\\ 0 & \sigma & -k_{2} & 0\\ 0 & \kappa & \gamma & -\mu \end{array}\right]\left[\begin{array}{c} E_{2}^{\left(1\right)}\\ E_{2}^{\left(2\right)}\\ E_{2}^{\left(3\right)}\\ E_{2}^{\left(4\right)} \end{array}\right]=-2\left[\begin{array}{c} G_{3}\\ -G_{3}\\ 0\\ 0 \end{array}\right].$$

It follows that

$$\begin{aligned} &E_{2}^{(1)} = \frac{2k_{1}k_{2}G_{3}}{(\mu+m_{1})k_{1}k_{2}-m_{2}k_{2}\mu-m_{3}\mu \sigma-m_{4}(\kappa k_{2}+\sigma\gamma)}, \\ &E_{2}^{(2)} = \frac{-2\mu k_{2}G_{3}}{(\mu+m_{1})k_{1}k_{2}-m_{2}k_{2}\mu-m_{3}\mu \sigma-m_{4}(\kappa k_{2}+\sigma\gamma)}, \\ &E_{2}^{(3)} = \frac{-2\mu\sigma G_{3}}{(\mu+m_{1})k_{1}k_{2}-m_{2}k_{2}\mu -m_{3}\mu\sigma-m_{4}(\kappa k_{2}+\sigma\gamma)}, \\ &E_{2}^{(4)} = \frac{-2(\kappa k_{2}+\sigma\gamma)G_{3}}{(\mu +m_{1})k_{1}k_{2}-m_{2}k_{2}\mu-m_{3}\mu\sigma-m_{4}(\kappa k_{2}+\sigma\gamma)}. \end{aligned}$$

Thus we can determine $W_{20}(\theta)$ and $W_{11}(\theta)$ from (5.20) and (5.21), respectively, and then we can compute $g_{21}$ by (5.14). Therefore we can compute the following values:

5.26 $$ \left. \begin{aligned}& c_{1}(0) = \frac{\text{i}}{2\tau_{n}\omega_{0}} \biggl(g_{11}g_{20}-2 \vert g_{11} \vert ^{2}-\frac{ \vert g_{02} \vert ^{2}}{3} \biggr)+\frac {g_{21}}{2}, \\ &\mu_{2} = -\frac{\operatorname{Re}\{c_{1}(0)\}}{\operatorname{Re}\{\lambda'(\tau _{n})\}}, \\ &\tilde{\beta}_{2} = 2\operatorname{Re}\bigl\{ c_{1}(0)\bigr\} , \\ &T_{2} = -\frac{\operatorname{Im}\{c_{1}(0)\}+\mu_{2}\operatorname{Im}\lambda'(\tau _{n})}{\tau_{n}\omega_{0}}. \end{aligned} \right\} $$

Based on our analysis, by the result of Hassard et al. [34] we have the following theorem.

### Theorem 5.1

*For delayed model* (2.1), *when* $\tau=\tau_{0}$, *the direction and stability of a periodic solution of Hopf bifurcation are determined by considering the signs of* $\mu_{2}$, $\tilde{\beta}_{2}$, *and* $T_{2}$, *respectively*, *given in* (5.26). *Then*

(i) *if* $\mu_{2}<0$ $(\mu_{2}>0)$, *then the Hopf bifurcation is subcritical* (*supercritical*) *and the bifurcation periodic solutions exist for* $\tau<\tau_{0}$ $(\tau>\tau_{0})$;
(ii) *if* $\tilde{\beta}_{2}>0$ $(\tilde{\beta}_{2}<0)$, *then the bifurcation periodic solutions are unstable* (*stable*);
(iii) *if* $T_{2}<0$ $(T_{2}>0)$, *then the period of the bifurcating periodic solutions decreases* (*increases*).

## Numerical simulations

To illustrate the dynamic behavior and the phenomenon of Hopf bifurcation of a delayed SEIR epidemic model, we integrate system (2.1) numerically by using the standard MATLAB algorithm with the parameter values/ranges in Table 1.

**Table 1:** Description of variables and parameters of the model

Parameter	Description	Value	Source
Π	Constant immigration rate	160 week^−1^	–
*β*	Contact rate	Variable	–
$\beta_{E}$	Ability to cause infection by exposed individuals $(0\leq \beta_{E}\leq 1)$	0.21	–
$\beta_{I}$	Ability to cause infection by infectious individuals $(0\leq \beta_{I}\leq 1)$	0.84	–
*μ*	Natural death rate	0.000263 week^−1^	[35]
*σ*	Duration of latency	2.32 week^−1^	[36–38]
*γ*	Recovery rate in infectious period	1.4 week^−1^	[36–38]
*κ*	Recovery rate in latent period	1.3 week^−1^	[39]
*α*	Disease-induced mortality rate	0.065 week^−1^	[40]

**Figure 1:**
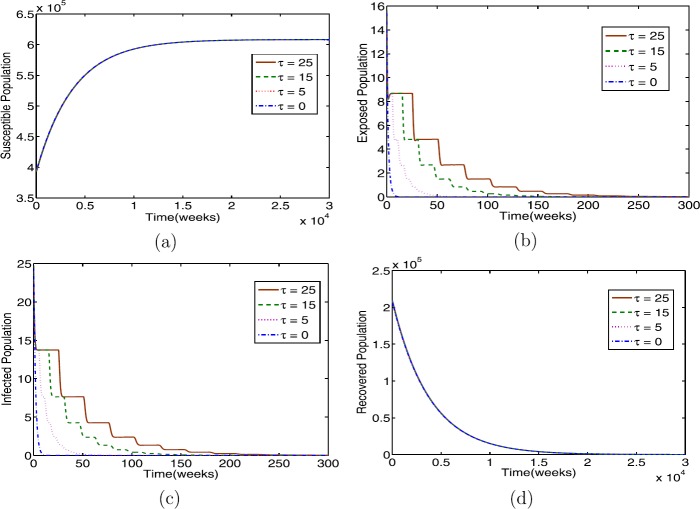
The profile solutions of delayed model (2.1) for $\tau = 0, 5,15,25$. The parameter values used in Table 1 and $\beta =2$ so that $R_{0} < 1$. The initial conditions are $S(0)=3.9327 \times 10^{5}$, $E(0)=15$, $I(0)=24$, and $R(0)=2.0894\times 10^{5}$

For parameters in Table 1 with $\beta= 2$, we have $R_{0} = 0.085<1$. As is evident from Fig. 1, whenever $R_{0} < 1$, the solution profiles converge to a disease-free equilibrium $\mathcal{E}_{0}$ for any chosen time delay *τ*, as in Theorems 3.1(i) and 3.2. By comparing with $\tau=0$, time delay has effect to the profiles of exposed and infectious individuals, making them oscillately converge as shown in Fig. 1(a), (d). On the other hand, the time delay has no impact on the profiles of susceptible and recovered individuals as *τ* increases; see Fig. 1(b,c). These results can be interpreted so that the disease is delayed and eventually extinct, that is, the disease disappears in the population.

**Figure 2:**
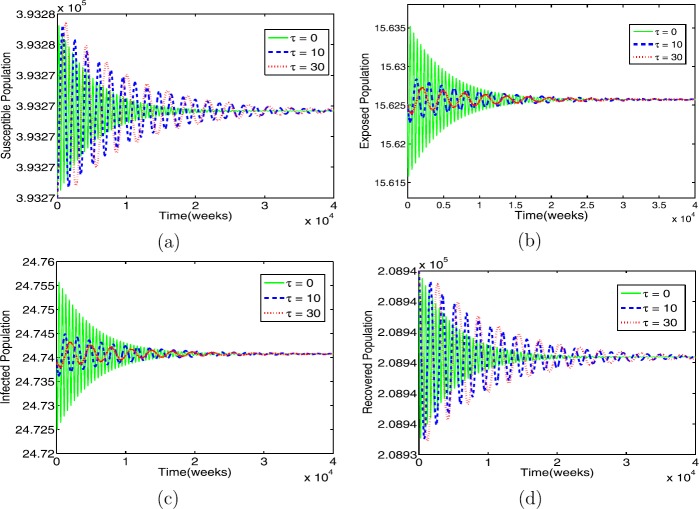
The profile solutions of delayed model (2.1) for $\tau = 0, 10,30$. The parameter values used in Table 1 and $\beta =3.6$ so that $R_{0} = 1.5314 > 1$. The initial conditions are $S(0)=3.9327 \times 10^{5}$, $E(0)=15$, $I(0)=24$, and $R(0)=2.0894\times 10^{5}$

In the case $R_{0} > 1$, the dynamics behavior of model (2.1) is explored with various contact rates and time delays. The contact rate *β* is chosen to be $\beta= 3.6$ and $\beta= 7.2875$. It is found that, when $\beta= 3.6$, the condition in Theorem 4.3(i) holds. It is seen that all solutions of model (2.1) converge to an endemic equilibrium $\mathcal{E}^{*}$ for all chosen *τ*; see Fig. 2. This verifies that the endemic equilibrium of (2.1) is absolutely stable, as guaranteed by Theorem 4.3(i). The results also show that the qualitative behavior of the model does not change as time delay increases.

Biologically, we observe that, as time delay increases, the numbers of exposed and infectious individuals decrease (see Figs. 2(b), (c)), whereas the numbers of susceptible and recovered individuals increase (see Figs. 2 (a,d)) due reduction in the chance of infection of susceptible individuals, and infectious population recovers from the disease (then they become members of the recovered group).

**Figure 3:**
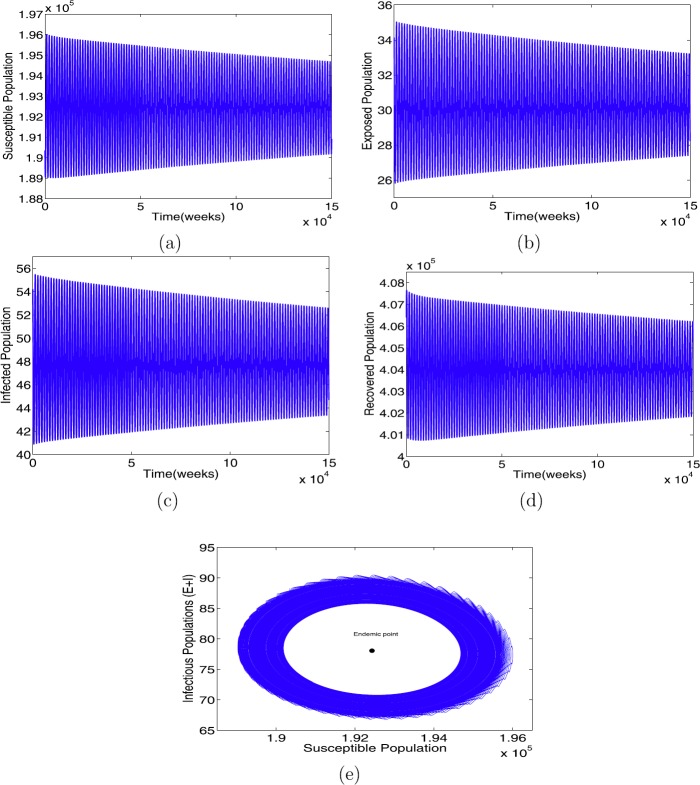
The profile solutions of delayed model (2.1) for $\tau = 25 < \tau_{0}$. The parameter values used in Table 1 and $\beta =7.2875$ so that $R_{0} = 3.1 > 1$. The initial conditions are $S(0)=1.92465\times 10^{5}$, $E(0)=30$, $I(0)=47$, and $R(0)=4.03998\times 10^{5}$

**Figure 4:**
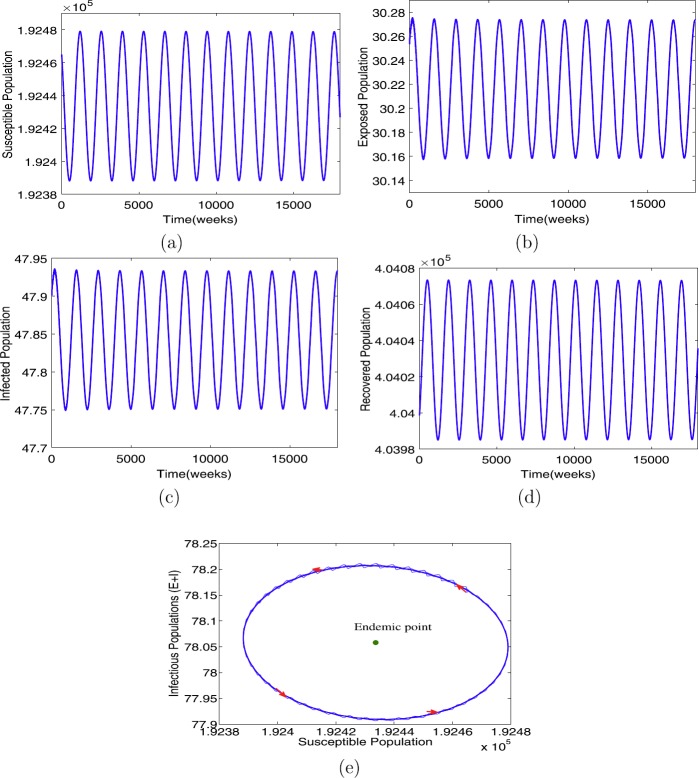
The profile solutions of delayed model (2.1) for $\tau = 25.86\approx \tau_{0}$. The parameter values used in Table 1 and $\beta =7.2875$ so that $R_{0} = 3.1 > 1$. The initial conditions are $S(0)=1.92465\times 10^{5}$, $E(0)=30$, $I(0)=47$ and $R(0)=4.03998\times 10^{5}$

**Figure 5:**
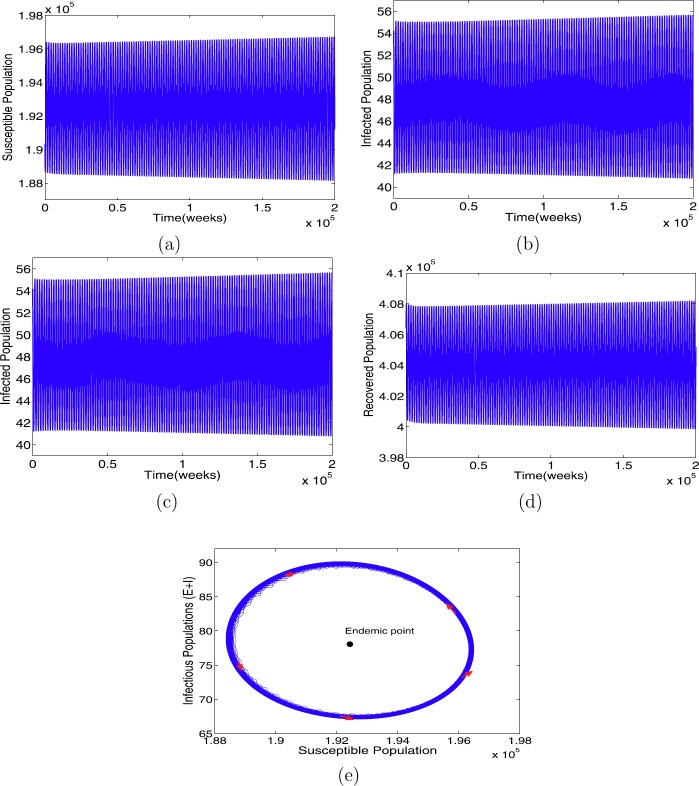
The profile solutions of delayed model (2.1) for $\tau = 35 > \tau_{0}$. The parameter values used in Table 1 and $\beta =7.2875$ so that $R_{0} = 3.1 > 1$. The initial conditions are $S(0)=1.92465\times 10^{5}$, $E(0)=30$, $I(0)=47$, and $R(0)=4.03998\times 10^{5}$

When $\beta= 7.2875$ with the other parameters in Table 1, the condition in Theorem 4.3(ii) holds. Further, we have $R_{0}=3.1$, an endemic equilibrium $\mathcal{E}^{*}=(1.9243\times10^{5},30.2160,47.8420,4.0403 \times 10^{5})$, and the critical time delay $\tau_{0}=25.86$. The solutions of model (2.1) as *τ* increases are illustrated in Figs. 3–5. We have found that $\mathcal{E}^{*}$ is asymptotically stable when $\tau= 25 <\tau_{0}$ (see Fig. 3), limit circle when $\tau\approx \tau_{0}$ (see Fig. 4), and asymptotically unstable when (see Fig. 5), respectively. Furthermore, we can calculate the following values: $c_{1}(0)=1.6548\times 10^{-13}-3.5212\times10^{-11}\text{i}, \mu_{2}=-4.6952\times10^{-7}, \tilde{\beta}_{2}=3.3096\times10^{-13}$, which verify that the endemic equilibrium $\mathcal{E}^{*}$ is asymptotically stable for $0< \tau<\tau_{0}$ (see Fig. 3); when $\tau\geq \tau_{0}$, $\mathcal{E}^{*}$ loses its stability (see Fig. 5), and a Hopf bifurcation occurs at $\tau\approx \tau_{0}$ (see Fig. 4), that is, a family of periodic solutions bifurcate from $\mathcal{E}^{*}$ (see Fig. 5), as guaranteed by Theorem 5.1.

In addition, we see that the critical time delay for Hopf bifurcation is a large number ($\tau_{0}=25.86$), which is realistic in the study of the effect of time delay in an epidemic model because adding a time delay in the model destabilizes the system and periodic solutions can arise through Hopf bifurcation, which impacts the effectiveness of disease control. If Hopf bifurcation, therefore, occurs at a large time delay, then the authorities involved with disease control may have enough time to act before the exposed individuals can become infective and infect other members of the population.

## Conclusion

This paper presents a delayed *SEIR* epidemic model with infectious force in latent and infected periods for studying the existence of Hopf bifurcation. The model is rigorously analyzed to gain insight into its dynamical features. The study results are summarized as follows. By using the Lyapunov functional method and the LaSalle invariance principle, the disease-free equilibrium is globally asymptotically stable if a certain threshold quantity, known as the reproductive number and denoted by $R_{0}$, is less than unity for all time delays $\tau\geq0$, indicating that time delay does not impact on the stability property of this equilibrium. When $R_{0}> 1$, the contact rate *β* and time delay *τ* are regraded as bifurcated parameters. The study results show that if the contact rate *β* satisfies condition (4.8), then the endemic equilibrium $\mathcal{E}^{*}$ of model (2.1) is absolutely stable, that is, $\mathcal{E}^{*}$ is asymptotically stable for all $\tau\geq0$. Meanwhile, if the contact rate *β* satisfies condition (4.9), then the endemic equilibrium, $\mathcal{E}^{*}$ of model (2.1) is conditionally stable, that is, $\mathcal{E}^{*}$ is asymptotically stable for $\tau\in[0,\tau_{0})$, and the Hopf bifurcation occurs at $\tau= \tau_{0}$. It is observed that the delayed *SEIR* epidemic model with infectious force in latent and infected period (2.1) exhibits a Hopf bifurcation, called subcritical, which is a different result from the epidemic models with bilinear incidence rate and nonlinear incidence rate that exhibit supercritical Hopf bifurcation; see [27, 41–44]. This gives the new result that the type of Hopf bifurcation depends on the type of incidence function used in the epidemic model. In addition, the phenomenon of Hopf bifurcation in the delayed *SEIR* epidemic model with infectious force in latent and infected period depends on contact rate in the sense that the contact rate is a crucial condition to ensure the Hopf bifurcation and time delay can cause the loss of stability via subcritical Hopf bifurcation at the critical time delay $\tau= \tau_{0}$.

In terms of disease control campaigns, this study result shows that the infection rate can be effectively controlled in a community if some public health measures are initiated that can reduce the contact rate. There exists an endemic equilibrium state, which is asymptotically stable, and the transmission of the disease seems to happen immediately (without any delay). Besides public health education, there is another way to destabilize this state and make the education more effective: by the management and care of exposed individuals in a timely fashion at the supervision or the direction of a legally qualified medical practitioner. So, delay in diagnosis and treatment of disease is one of the reasons for the failure in the control of the disease.
